# Drug-induced interstitial lung disease: mechanisms and best diagnostic approaches

**DOI:** 10.1186/1465-9921-13-39

**Published:** 2012-05-31

**Authors:** Osamu Matsuno

**Affiliations:** 1 Division of Medicine for Allergic Disease, Osaka Prefectural Medical Center for Respiratory and Allergic Diseases, 3-7-1 Habikino, Habikino City, Osaka, 583-8588, Japan

**Keywords:** DILD, Drug-induced interstitial lung disease, DPT, drug provocation test, DLST, Drug-induced lymphocyte stimulation test, LMT, leukocyte migration test

## Abstract

Drug-induced interstitial lung disease (DILD) is not uncommon and has many clinical patterns, ranging from benign infiltrates to life-threatening acute respiratory distress syndrome. There are two mechanisms involved in DILD, which are probably interdependent: one is direct, dose-dependent toxicity and the other is immune-mediated. Cytotoxic lung injury may result from direct injury to pneumocytes or the alveolar capillary endothelium. Drugs can induce all types of immunological reactions described by Gell and Coombs; however, most reactions in immune-mediated DILD may be T cell-mediated.

DILD can be difficult to diagnose; diagnosis is often possible by exclusion alone. Identifying the causative drug that induces an allergy or cytotoxicity is essential for preventing secondary reactions.

One method to confirm the diagnosis of a drug-induced disease is re-exposure or re-test of the drug. However, clinicians are reluctant to place patients at further risk of illness, particularly in cases with severe drug-induced diseases. Assessment of cell-mediated immunity has recently increased, because verifying the presence or absence of drug-sensitized lymphocytes can aid in confirmation of drug-induced disease. Using peripheral blood samples from drug-allergic patients, the drug-induced lymphocyte stimulation test (DLST) and the leukocyte migration test (LMT) can detect the presence of drug-sensitized T cells. However, these tests do not have a definite role in the diagnosis of DILD. This study explores the potential of these new tests and other similar tests in the diagnosis of DILD and provides a review of the relevant literature on this topic.

## Introduction

Several types of drugs can cause drug-induced interstitial lung disease (DILD). The incidence of DILD for each individual drug is variable. DILD may be mild to progressive. In its more severe manifestation, DILD may result in respiratory failure and acute respiratory distress syndrome. DILD may develop within the first few days of treatment or may not until several years after treatment. DILD is generally described in terms of its clinical/histopathological features. The mechanisms involved in drug-induced lung injuries are unclear; therefore, DILD cannot be classified in terms of pathogenesis.

Diagnosis of DILD generally depends on a definite temporal association between an exposure to the causative agent and the development of respiratory signs and symptoms. The most important for accurate diagnosis is the exclusion of other causes of lung damage. Specific markers, histological findings, and diagnostic clinical features are generally unremarkable in DILD [1,2]. Difficulties arise when signs and symptoms develop after the drug is discontinued rather than during treatment or when no improvement follows discontinuation of the drug. Making a timely and accurate diagnosis of DILD is very important to ensure a favorable outcome [3].

## Clinical manifestations and diagnosis of DILD

### Clinical manifestations

Laboratory findings and clinical manifestations of DILD, such as cough, fever, dyspnea, and hypoxemia [1-4], are non-specific. DILD is indicated when cough, fever, dyspnea, and/or pleuritic chest pain are observed in combination with pertinent radiographic findings, in the absence of evidence of congestive heart failure, infectious disease or malignancy, and when symptoms subside with drug withdrawal. Pulmonary function tests in the majority of DILD cases may reveal a pattern of restrictive abnormality, with decreased values of DLCO. Discontinuation of the drug is essential, and, in more severe cases, administration of corticosteroids may be of therapeutic value.

### Histology

The histological findings of pulmonary drug reactions are often non-specific and mimic those of other conditions, such as idiopathic interstitial pneumonia and collagen vascular disease [5]. Almost, all histopathological subtypes of interstitial lung disease may be observed [3]: diffuse alveolar damage (DAD), chronic interstitial pneumonia [CIP, including non-specific interstitial pneumonia (NSIP), usual interstitial pneumonia (UIP), and desquamative interstitial pneumonia (DIP)], organizing pneumonia (OP), eosinophilic pneumonia (EP), hypersensitivity pneumonitis (HP), and granulomatous lung disease [6-9]. While some drugs, such as minocycline, methotrexate (MTX), and nitrofurantoin, induce stereotypical reactions in the lungs (EP, acute granulomatous interstitial lung disease, and the cellular type of non-specific pneumonia, respectively), other drugs, such as amiodarone and bleomycin, may be associated with more than one histological pattern [3].

### High-resolution computed tomography (HRCT) and ^18^ F-fluorodeoxyglucose positron emission tomography (^18^ F-FDG-PET)

HRCT is currently the best non-invasive method to assess the presence of drug-induced lung disease. The results of HRCT in DILD are similar to those of ILDs from other causes or those of idiopathic interstitial pneumonia. Radiographic manifestations of DILD correspond to those of NSIP, UIP, HP, DAD, cryptogenic OP (COP)/EP, and diffuse pulmonary hemorrhage. HRCT may reveal abnormalities in patients with normal radiographs [8,10,11]. Cleverly et al. reported that HRCT had an accuracy of only 45% for predicting the specific histological reaction pattern of DILD [5]. Thus, HRCT is limited in its ability to predict the histological patterns in drug-induced lung diseases [5]. HRCT can, however, be valuable in identifying findings that suggest an alternative diagnosis and in monitoring responses to treatments.

Akira et al. reported difuuse or multi-focal ground-glass opacities with intralobular interstitial thickening as the predominant findings in anti-neoplastic agent-induced pneumonitis. Patchy ground-glass opacities with centrilobular opacities and interlobular septal lines were predominant radiographic findings in antibiotic agent-induced pneumonitis [12].

Recently, the use of ^18^ F-FDG-PET in the diagnosis of DILD has been reported. FDG uptake was detected at an extremely early stage when no symptoms or abnormal findings on HRCT were apparent [13,14]. PET positivity, however, has no specificity for DILD.

### Serum markers

KL-6 has been reported as a sensitive marker for interstitial lung diseases [4,15]. Particular patterns detected by HRCT, such as DAD and CIP, but not COP/EP or HP, are associated with increased KL-6 levels in the circulation of patients with DILD [6]. Furthermore, surfactant proteins SP-A and SP-D have been reported as specific markers of pulmonary fibrosis [16]. Inomata et al. demonstrated that SP-A, SP-D, and KL-6 levels were increased in the serum of patients with DILD associated with the administration of the epidermal growth factor receptor-tyrosine kinase inhibitor, gefitinib [17].

Serum ADAM8 (a disintegrin and a metalloproteinase 8) concentrations were significantly elevated in patients with drug-induced EP, and after a drug provocation test (DPT), which demonstrated that ADAM8 induction paralleled drug-induced eosinophilic lung inflammation [18].

### Bronchoscopy and bronchoalveolar lavage (BAL)

Bronchoscopy can be helpful in determining the presence of pneumonitis and for the differential diagnosis of lymphangitic carcinomatosis, vasculitis, alveolar hemorrhage, or pneumonia from infectious agents.

Most drug-induced immunological reactions, such as HP and COP/EP, may be excluded if BAL cytology is normal. The most prominent feature of DILD was a lymphocytic alveolitis, either pure or associated with neutrophil and/or eosinophilic alveolitis along with an imbalance in T lymphocyte phenotype [19]. The most frequent change observed is lymphocytic alveolitis with a preponderance of CD8+ cells. In MTX-induced pneumonitis, CD4+ cells may also be preferentially increased; this increase has also been demonstrated with the use of ampicillin, nitrofurantoin, and sirolimus [20].

## Mechanisms involved in DILD

Many different mechanisms may be involved in the initiation and propagation of DILD [21]. Both cytotoxic and immune mechanisms of action may be involved independently or in combination in the tissue expression of different forms of lung injury [22].

### Cytotoxic pulmonary injury

Multiple mechanisms may be responsible for cytotoxic pulmonary injury due to drugs, including reactive oxygen species (ROS) [21,23-25], reduction in deactivation of metabolites of the lung [26-28], impairment of alveolar repair mechanisms [29-31], and release of various cytokines [23]. Many agents may be toxic to the lungs. These include cytotoxic drugs, such as bleomycin, MTX, and cyclophosphamide, and non-cytotoxic drugs, such as nitrofurantoin, sulfasalazine, and amiodarone [32].

Chemotherapy lung is one representative example of cytotoxic lung injury. It is a severe type of pulmonary reaction that develops during or shortly after treatment with chemotherapeutic agents, such as antibiotics, alkylating agents, anti-metabolites, nitrosamines, rapamycin analogs, and podophyllotoxins [4]. Histologically, chemotherapy lung corresponds to DAD [1,3]. Concurrent radiation or oxygen therapy increases the risk of developing chemotherapy lung. Moreover, chemotherapy lung can sometimes develop because of previously unresolved chemotherapy- or radiation-induced damage with additional chemotherapy [3].

#### Mechanisms of cytotoxic pulmonary injuries

The pathogenesis of cytotoxic lung injury may include direct injury to pneumocytes or the alveolar capillary endothelium, with subsequent release of cytokines and recruitment of inflammatory cells. The systemic release of cytokines induced by chemotherapeutic agents (e.g., gemcitabine) may also result in capillary leakage and pulmonary edema. Early events in lung injury induced by tricyclic antidepressants may be related to endothelial damage [21] because of impaired tight junctions mediated by amitriptyline-induced perturbations in intracellular calcium [21].

MTX-induced pulmonary toxicity may induce the release of free oxygen radicals, such as nitric oxide, and various cytokines, such as IL-1β, TNF-α, and TGF-β. Kim et al. reported that the p38MAPK signaling pathway was associated with a pulmonary inflammatory response [23].

By impairing alveolar repair mechanisms, gefitinib may potentiate the effects of lung injury [29,30]. Suzuki et al. have suggested that gefitinib therapy may augment any underlying pulmonary fibrosis through decrease in epidermal growth factor receptor phosphorylation with coincident regenerative epithelial proliferation [31].

The toxic mechanism of amiodarone leads to the disruption of the lysosomal membranes of molecules through protein C activation and the subsequent release of toxic oxygen radicals, which may induce activation of caspase pathways and lead to apoptosis of lung epithelial cells [24]. An additional mechanism reduces deactivation of toxic metabolites of the drug [26,27].

Pulmonary toxicity may also be caused by the generation of free oxygen radicals by mitomycin C, nitrofurantoin, and bleomycin. These drug-induced ROS generate substances such as H_2_O_2_, O_2_-, and OH [21]. In vivo and in vitro studies showed bleomycine, a cancer chemotherapeutic agent, to be the cause of pulmonary toxicity, which was mediated, at least partly, by a bleomycin-iron complex, generating toxic O_2_-derived species within the lung [25]. Particular susceptibility to bleomycin toxicity in the lung may depend on the fact that bleomycin is preferentially distributed in lung tissue and that the lung is relatively deficient in the hydrolase enzyme that detoxifies bleomycin [28].

Nitrofurantoin and bleomycin share the ability to generate O_2_ radicals and to cause lung damage. The reason that these drugs affect the lungs as their predominant site of toxicity remains unclear. One possibility is the rate of gas exchange and high oxygen load in the lungs, which enables damage due to these drugs [21].

#### Diagnosis of cytotoxic pulmonary injury

Drug-induced pulmonary toxicity can be difficult to diagnose because cancer patients are usually administered multiple anti-neoplastic agents; thus, identifying the causative agent becomes difficult. Unfortunately, no single diagnostic test or tissue biopsy is currently available that can definitively confirm a diagnosis of chemotherapy-associated lung disease [33].

Currently, in vitro drug challenge is not a readily available or clinically validated diagnostic assay for cytotoxic lung injury. Reactive drug metabolites are believed to play a role in many drug reactions. Differences in the capacity of cells to detoxify the reactive metabolites of drugs are important determinants in drug toxicity reactions, and these differences could be used as the basis of a diagnostic assay. Microsomes are a source of oxidative enzymes, primarily cytochrome P450 (CYP). Cell viability can be determined after incubating microsomes with PBMCs and the suspected drug in the presence or absence of a microsomal activating system. This assay has not been used for the diagnosis of cytotoxic lung injury. However, this approach may enhance our understanding of selected drugs that cause DILD, paving the way for development of clinically useful assays [34,35].

### Immune-mediated pulmonary injuries

#### General pathogenesis of drug allergies

Exposure to a particular drug can induce immune reactions in a minority of individuals. Although most have not yet been identified, several factors may control this process: the chemical structure of the molecule (antigenic drug determinants); genetic factors including polymorphisms of genes that encode drugs that metabolize enzymes and immune responses; environmental factors (stress, concurrent infection, and pollution); and the nature of drug exposure (dose, duration, frequency, and route of administration) [36].

#### (a) mechanisms of drug allergy

Our knowledge regarding human immune responses to drugs remains limited. Drug hypersensitivity and other immune reactions are frequently categorized according to Gell and Coombs classification, which includes four categories that reflect distinct immune mechanisms. This variety explains the heterogeneous clinical presentations that can occur [37]. All these reactions are T cell-regulated, but the effector functions are primarily antibody-mediated factor functions (I-III). Most of the reactions in immune-mediated DILD may be T cell-mediated. DILD associated with a type III reaction was investigated in a case report. Schatz et al. reported a case of pneumonitis characterized by an eosinophilic interstitial infiltrate with evidence of immunofluorescent staining and immune complex-mediated activation of the classic complement pathway [38].

#### (b) drug recognition

With regard to the role of T cells in drug hypersensitivity, a question arises: how do T cells recognize drugs? [34].

#### (b)-(i) hapten concept

The recognition of small molecules like drugs by B and T cells is usually explained by the hapten concept. In their native states, most drugs are not immunogenic. Many drugs have low molecular weights (<1000 kDa) and must be covalently bound to high-molecular-weight carrier proteins to become effective immunogens [36,39]. A hapten may directly bind to an immunogenic peptide presented by a major histocompatibility complex molecule.

A drug that is not chemically reactive per se may become reactive on metabolism [39]. Once formed, these reactive metabolites may cause cytotoxicity and/or become covalently bound to cellular proteins [40]. The ability to form immunogenic complexes and mount an immune response to these complexes differs among individuals [39].

#### (b)-(ii) p-i concept

Pichler et al. elaborated another possibility; namely, a pharmacological interaction between drugs and immune receptors (p-i concept) [36]. They found that chemically inert drugs, which could not covalently bind to peptides or proteins, could directly activate certain T cells if they happened to bear T cell receptors that could interact with the drug. According to the “p-i” model, which is metabolism- and processing-independent, the structural features of a drug elicit an immunological response [36].

#### (b)-(iii) danger model

One of the major functions of the adaptive immune system is to distinguish “self” from “non-self.” If the immune system encounters self proteins, it will result in tolerance. In contrast, encounter with non-self or foreign proteins leads to an immune response.

Matzinger proposed an alternative explanation, labeled the Danger Model, of the generation of immune responses [40]. According to Matzinger, the activation of dendritic cells can be induced by endogenous danger signals, such as - release by tissues undergoing stress, damage, or abnormal death, and by exogenous danger signals elaborated by pathogens. Certain drugs may cause cell injury and act as immunologic triggers.

**Figure 1:**
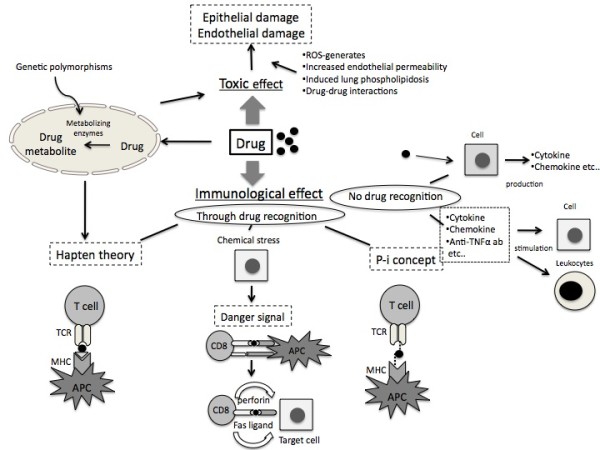
Schematic representation of drug-induced pulmonary effects and toxicity including hypothetical pathogenenic mechanisms involved.

A schematic representation of drug-induced pulmonary effects and toxicity is provided in Figure 1.

#### Diagnostic tests in drug hypersensitivity reactions

In vitro diagnostic tests are useful in cases of clinical uncertainty to confirm a clinical diagnosis. To determine which test is optimal for confirming the clinical diagnosis of an adverse drug reaction, an appreciation of the pathophysiological mechanism(s) responsible for the adverse reaction is critical [34]. Potentially useful diagnostic tests in DILD include the drug lymphocyte stimulation test (DLST), LMT [41], and flow cytometry.

### Mechanisms and diagnosis of immune-mediated DILD

Lungs provide a barrier to illness in which the immune system is chronically activated to provide optimal host defense. This constant, low-level activation provides a milieu that may facilitates pro-inflammatory signals and subsequent immune system responses.

Minocycline-induced pneumonia (MIP) is generally manifested as EP. A central role for T lymphocytes in the immunologic reaction to MIP was suggested by Gillon et al., who identified lymphocyte-mediated specific cytotoxicity against minocycline-bearing alveolar macrophages in vitro [42]. Their findings, however, do not explain the presence of pulmonary eosinophilia, which is a characteristic feature of MIP. Thus, further investigations are needed to elucidate the pathophysiology of MIP. Three different mechanisms of amiodarone induced lung disease have been suggested: a direct toxic effect, an immune-mediated mechanism, the angiotensin enzyme system activation [9]. From the immunological perspective, Kuruma et al. reported that the Th1/Th2 balance may influence amiodarone metabolism and may be a powerful indicator of amiodarone-induced subclinical lung toxicity [43].

### (*a*) diagnosis of immune-mediated DILD

Causative drugs are determined from the history of drug exposure and any response to withdrawal of an implicated drug. An in vitro test such as the DLST can only detect the presence of sensitization, but cannot predict whether the sensitization will lead to symptoms [44]. Provocation tests can detect not only the presence of sensitization to a drug, but also the clinical manifestations induced by this sensitization [44]. The utility of skin testing in DILD, however, has not been reported.

### (*a*)-(i) in vitro tests for DILD

DLST and LMT are bioassays that help to confirm the presence of drug-sensitized lymphocytes. The DLST verifies the growth of sensitized lymphocytes after a drug is used for antigen stimulation, while the LMT identifies the cytokines or chemokines produced by sensitized lymphocytes after a drug is used for antigen stimulation [45].

The DLST is the most commonly used in vitro test for detecting the causative drug in cases of drug allergy. The causes of drug allergies determined using the DLST have been extensively reported in cases of drug eruption [44,46,47]. Laboratory-based in vitro methods, such as the DLST, have numerous advantages, including absolute safety, ability to assess T cell responses to multiple drugs simultaneously, and absence of risk of developing additional drug allergies [44]. This technique measures the uptake of a DNA precursor (tritiated ^3^ H] thymidine) after lymphocytes have been exposed to an antigen in vitro. This test is associated with blast formation by lymphocytes [44].

The LMT demonstrates the presence of sensitized lymphocytes when granulocytes, either alone or mixed with normal lymphocytes, exhibit migration inhibition when cultured at optimal drug concentrations. Saito et al. reported that the LMT had a higher positive response rate than the DLST for several hypersensitivity symptoms, such as skin eruptions and hepatic injury [45].

While DLST has been widely used in the diagnosis of DILD in Japan, this is not the case in other countries. Compelling data as to the sensitivity and specificity of the DLST for DILD is currently lacking. A cell-mediated hypersensitivity reaction on DLST is the basis for diagnosis of gold-induced pneumonia (GIP). Tomioka and King included CD8+ lymphocytic alveolitis and a positive DLST in their diagnostic scheme for GIP [39]. However, GIP is rarely a clinical problem at the present time. Studies on the use of DLST in DIP are far from well controlled. Based on data that compared the results of the DLST and provocation tests for patients with DILD, DLST contributed little in detecting the causative agents in these patients [48,49].

DLST is believed to be insensitive to MIP. Toyoshima et al. reported the results of DLST for minocycline in six patients; in all cases, the results were negative [50]. In addition, suppressive effects of minocycline on T cell proliferation have been described [51,52].

For MTX and Kampo (Japanese herbal) drugs, the results of DLST tend to be overestimated. Many reports have provided evidence that the uptake of ^3^ H thymidine into lymphocytes in the presence of MTX may be explained by the upregulated incorporation of thymidine from the extracellular space following the depletion of the intracellular thymidine pool caused by MTX. In one study, MTX created an early block in the cell cycle without reducing the cellular uptake of ^3^ H-thymidine [53]. Hoffman also found a discrepancy between the formation of blast-like cells and high thymidine uptake in MTX-treated, mitogen-stimulated lymphocytes [54]. Hirata et al. showed that DLST using MTX was inadequate in confirming MTX-induced DILD [55].

Kampo drugs are generally contaminated with non-specific mitogens from plants. Mantani et al. reported positive DLST results for Kampo drugs in 85.7% of enrolled patients not taking any Kampo medicines [56]. In addition, several studies reported discrepancies between DLST findings and results of provocation drug tests in Kampo drugs [7,18,48,49].

Leukocyte migration inhibition factor production has also been observed in well-established cases of hypersensitivity pneumonitis, such as that due to beryllium [42,57]. Various reports have drawn attention to the positive results of this test in cases of DILD due to different drugs, such as minocycline, amiodarone, propranolol, nitrofurantoin, gold salts, MTX, and paclitaxel [41,57-61]. However, the number of samples used in these reports were small.

CD69 upregulation on T cells has been reported as an in vitro marker for delayed-type drug hypersensitivity reactions [62]. Compared with other early T cell activation markers, such as CD71 or CD25, CD69 appeared to be most suitable, as it was rapidly upregulated and showed the greatest difference from baseline values [63].

CD69 upregulation was shown to be drug-specific and not an inherent property of cells derived from patients. It did not occur in unstimulated cultures or in drug-stimulated PBMCs from non-allergic donors. Cells from drug-allergic subjects reacted only to the causative drug, but not to any other tested drugs [63]. This assay has not been used for the diagnosis of DILD. However, this approach may enhance our understanding of selected drugs that cause DILD.

### (*a*)-(ii) drug provocation tests (DPT)

Rechallenge of patients with DILD is generally considered unethical as the pulmonary damage caused by DILD is largely irreversible, and re-challenge increases the risk to the patient. Re-test of drugs has been safely performed in some patients, although a fatality was reported following a re-test with MTX in one case [64]. Several investigators have conducted re-tests with minocycline to establish an accurate diagnosis [65] with no reported morbidity. Similar findings have been reported for EP induced by other drugs, such as sulfalazine [3], amoxicillin [7], and nimesulide [66].

Recently, Yasui et al [48]. proposed a DPT method for mild DILD. DPT was performed in 58cases, 41 of which showed positive results. This test was initiated by administering the lowest dosage of the suspected causal drug that could achieve a response, and then gradually increasing the dose at daily intervals until a normal daily dose was reached or symptoms occurred. This DPT protocol was deemed useful according to the criteria provided by Yasui et al [48,49]. Their diagnostic criteria for DIP included a 1°C increase in body temperature and one or more of the following an increase in the alveolar-arterial difference in oxygen tension (A-aDO2) of >10 mmHg; an increase >20% in white blood cell count; and positive conversion of C-reactive protein [48,49].

## Pulmonary inflammation and fibrosis

Monoclonal antibodies have emerged as a new class of agents that can cause DILD. The number of cases of ILD triggered by anti-TNF-α agents is increasing. TNF-α has both pro- and anti-fibrotic effects. TNF-α can promote pulmonary tissue repair, eliminating inflammatory cells by inducing their apoptosis [52,67,68]. Increase in the local releases of inflammatory cell-derived proteolytic enzymes may enhance the potential pulmonary toxicity of MTX, which may then be potentiated by an anti- TNF-α agent, resulting in impaired apoptosis of infiltrating inflammatory cells [67]. Anti- TNF-α therapy may also result in systemic and/or pulmonary shifts towards anti-inflammatory cytokines, such as TGF-β1, thus contributing to a pro-fibrotic state [67], which would explain the exacerbation seen in pulmonary fibrosis.

Rituximab, an anti-CD20 antibody, has been reported to induce a heterogeneous spectrum of lung disease. The release of cytokines, such as TNF-α, IFN-α, IL-6, and IL-8, has been postulated as the mechanism responsible. Other possible mechanisms of induction include complement activation or indirect cytotoxic T lymphocyte (CTL) activation [69]. CTL activation appears to be induced by dendritic cells that, in turn, are stimulated by cell-derived peptides resulting from rituximab-induced cell destruction. These activated CTL’s may cause vascular and alveolar damage thereby, initiating lung injury [14,69].

## Drug interactions and cross-reactivity

Drug interactions that cause ILD have not been reported except in one case report by McFadden et al., who reported a case of “gold-naproxen pneumonitis.” They hypothesized that naproxen interfered with the ability to restrain the immune response to gold, which resulted in clinical pneumonitis that resolved only when both naproxen and gold were discontinued. This was the first suggestion of a “drug interaction” that could cause lung injury [70].

Cross-reactivity has not been documented with drugs that cause ILD [3].

## Host susceptibility in DILD

The mechanisms by which some patients exhibit high susceptibility while others appears to be resistant remain largely unknown. However, several possibilities have been suggested.

The human leukocyte antigen (HLA) complex plays a central role in presenting antigens for T cell recognition. In certain immune based disorders, the pattern of HLA class II allele presentation confers both resistance and susceptibility to disease onset. Rheumatoid arthritis (RA) patients who tested positive for HLA-B40 had a 10.5-fold relative risk and those positive for Dw1 had a 6.2-fold relative risk of developing gold-induced pneumonitis as compared with RA patients without these antigens [71].

Both genetic and intrinsic factors can affect the disposition of certain drugs (absorption, distribution, metabolism, and excretion). Several different xenobiotic-metabolizing CYP and conjugation enzymes have been shown to be present in the lung [72-74].

CYP single nucleotide polymorphisms are among the key factors known to cause variations in drug responses among individuals [75]. Wijnen et al. indicated that DILD might be attributed to a reduced metabolic capacity because of CYP enzymes. They indicated that various CYP genotypes presented a substantial susceptibility risk factor for the development of DILD [76]. Therefore, genotyping prior to drug prescription may be clinically useful for predicting and preventing DILD.

## Conclusion

This review has focused on the mechanistic aspects of DILD, describing several different mechanisms that may be involved in the initiation and propagation of DILD. With better clarification of these processes, the prediction of DILD should be possible in the not too distant future. In addition, the validation of previously developed techniques and the development of new tests will contribute enormously to progress in the diagnostic evaluation of DILD.

## Competing interests

The author declares that he has no competing interest.
